# Sensitive and Discriminative Fluorescent Detection of Volatile Primary Aliphatic Diamine Vapors from Monoamines

**DOI:** 10.3390/molecules29245947

**Published:** 2024-12-17

**Authors:** Agostino Attinà, Ivan Pietro Oliveri, Massimiliano Gaeta, Santo Di Bella

**Affiliations:** Dipartimento di Scienze Chimiche, Università di Catania, Viale Andrea Doria 6, 95125 Catania, Italy; agostino.attina@phd.unict.it (A.A.); ivan.oliveri@unict.it (I.P.O.); massimiliano.gaeta@unict.it (M.G.)

**Keywords:** Zn(II) Schiff-base complex, fluorescence quenching, sensing aliphatic diamine vapors, paper-based sensor

## Abstract

The discriminative detection of volatile primary aliphatic diamines (VPADs) is a relevant and timely issue. This paper explores the distinctive optical features of H-type and J-type aggregates on paper-based (PB) films, namely H-PB and J-PB films, respectively, of a Lewis acidic Zn(salen)-type complex upon chemisorption of vapors of ditopic VPADs versus those of monotopic volatile amines. While volatile monotopic Lewis bases upon chemisorption give rise to mono-adducts accompanied by enhancement of the fluorescence, in contrast, VPADs act as ditopic bases forming di-adducts with distinct optical properties, leading to fluorescence quenching. This behavior enables the sensitive and discriminative detection of VPAD vapors from those of volatile monoamines. For example, for ethylenediamine (EDA), using J-PB films, sensitive detection is achieved with a LOD down to 6.6 ppm, lower than the OSHA permissible exposure limit of 10 ppm for EDA, and a linear dynamic range up to 100 ppm. Instead, H-PB films enable the detection of EDA vapors at higher ppm concentrations (up to 3000 ppm) with a linearity of up to 1000 ppm. Thus, the combination of both H-PB and J-PB films of the Zn(salen)-type complex represents a unique example of the sensitive and discriminative detection of EDA vapors in such a wide concentration range.

## 1. Introduction

Volatile aliphatic amines are an important class of organic bases involved in many applications, as raw materials or intermediate products in the manufacturing of other chemicals, pharmaceuticals, etc. [1]. Among them, a significant family is represented by the volatile primary aliphatic diamines (VPADs). For example, 1,2-diaminoethane or ethylenediamine (EDA) is the simplest aliphatic diamine and an important industrial chemical raw material, widely used in pesticides and fine chemical intermediates [2,3,4,5]. Its highly corrosive and toxic properties may put humans at risk when exposed to EDA vapors even at low concentrations [6]. In particular, for EDA, the Occupational Safety and Health Administration (OSHA) has established a permissible exposure limit (PEL) of 10 ppm, calculated as an 8 h time-weighted average [7]. Other members of the VPADs, such as cadaverine and putrescine, represent among the most important biogenic amines [8]. They are mainly formed in the tissues of living organisms by enzymatic decarboxylation of amino acids. Biogenic amines are present in fresh food, particularly in protein-rich samples, and their levels may rise due to improper storage [9]. Although almost all biogenic amines are involved in some specific physiological roles in living organisms, excessive amounts can result in toxicity [10,11]. For all these reasons, the discriminative detection of VPAD vapors from those of monoamines is a relevant issue for several applications, such as industrial and environmental monitoring [12,13] and food safety [14,15].

Optical chemosensors have been extensively used for the detection of aliphatic amine vapors [16,17,18,19,20,21], including vapochromic detection [22,23,24,25,26,27,28], mainly exploiting their basic properties. In this context, an important issue is the capability to distinguish and sensitively detect volatile aliphatic diamine vapors from monoamines. In this regard, several recent contributions have been reported for the optical detection of volatile aliphatic diamine vapors [29,30,31,32,33,34,35,36], some focused on their discriminative detection [37,38,39,40,41,42,43,44,45,46], while a few examples also concern their sensitive detection [47,48,49,50].

Zn(salen)-type Schiff-base complexes are a family of molecular materials characterized by the Lewis acidic character of the metal center [51], leading to a variety of molecular aggregates and supramolecular structures [52,53]. They can interact with Lewis bases forming stable Lewis acid-base adducts [54,55,56]. The latter possess distinct photophysical properties compared to the starting complexes. Therefore, these molecular materials have been developed as optical chemosensors of Lewis bases [57,58,59]. Their Lewis acidic character is also exhibited in the solid state, with very interesting vapochromic characteristics, upon the reversible chemisorption/desorption of Lewis base vapors [60,61,62,63], and responsive properties [64,65,66,67,68,69,70].

**Scheme 1:**
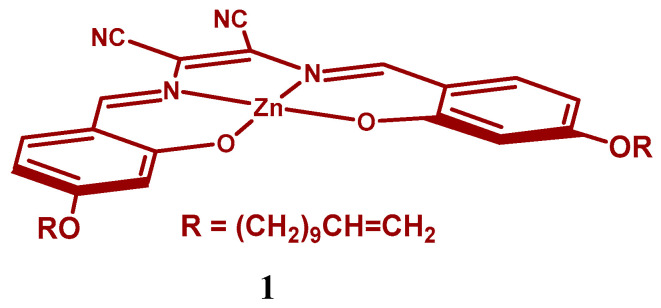
Structure of the complex **1**.

We have recently found that aggregates of the Zn(salen)-type complex, **1** (Scheme 1), are highly selective and sensitive to vapors of primary aliphatic amines, resulting in a substantial fluorescence enhancement upon chemisorption of these volatile organic compounds (VOCs) [71]. The formation of stable mono-adducts after deaggregation is responsible for the observed vapoluminescence.

To further investigate the vapochromic properties of this complex towards other classes of VOCs, we considered ditopic Lewis bases, such as VPADs, NH_2_-(CH_2_)*_n_*-NH_2_ (*n* = 2–5), and explored whether they behave differently by virtue of their ditopic character. We then investigated the possibility that these volatile ditopic molecules interact differently with aggregates of **1**, resulting in species having different optical properties than mono-adducts, thus distinguishing them from volatile monoamines.

In this paper, we demonstrate that paper-based (PB) films of complex **1** respond discriminatively to VPAD vapors from those of monoamines, especially to EDA, by fluorescence quenching enabling their sensitive detection.

## 2. Results and Discussion

### 2.1. Optical Response of PB Films to EDA Vapors

**Figure 1:**
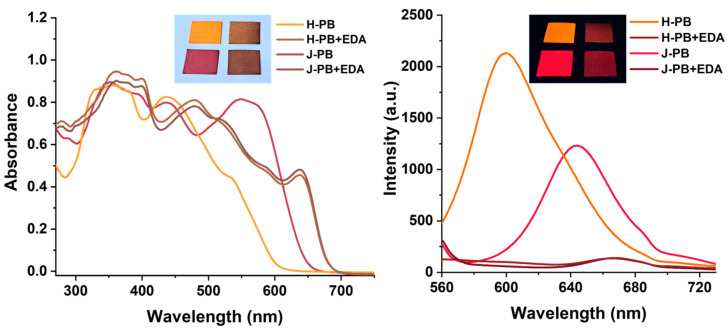
UV-vis reflectance (**left**) and fluorescence ((**right**); λ_exc_ = 460 nm) spectra of H-PB and J-PB films and after exposure to saturated EDA vapors. Inserts: photographic images of H-PB and J-PB films and after exposure to saturated EDA vapors under natural light (**left**) and 365 nm light (**right**).

Solid complex **1** exhibits a thermochromic behavior, characterized by two different structures. The dried pristine red solid forms H-type aggregates which, upon heating to 140 °C, are involved in a thermally induced irreversible phase transition with the formation of brown J-type aggregates [63]. The vapochromic properties of these aggregates can be properly explored on PB films, by depositing the complex onto paper substrates using the dip-coating technique. The as-prepared PB H-type aggregate (H-PB) films and the annealed PB J-type aggregate (J-PB) films of **1**, obtained by heating H-PB films to 140 °C, are characterized by different optical properties (Figure 1). The orange H-PB films show a broad absorption band in the visible region, ranging from 400 to 580 nm, centered at ca. 440 nm, and a strong fluorescence emission band centered at 600 nm. The red-brown J-PB films, conversely, show a broad absorption band centered at 560 nm and a fluorescence emission, less intense than that of H-PB films, at 644 nm.

Among VPADs, EDA was chosen as the prototype species. After exposure to saturated vapors of EDA (13,824 ppm at 23 °C) both H-PB (10 min exposure) and J-PB (5 min exposure) films turn dark brown, characterized by almost identical reflectance spectra, with a broad absorption in the 400–600 nm region and the formation of a new band at 638 nm, and by a complete quenching of their original fluorescence, at 600 nm and 644 nm for H-PB and J-PB films, respectively, along with the appearance of a new very weak fluorescence emission at ca. 665 nm (Figure 1). Therefore, the interaction of H-PB and J-PB films of **1** with EDA vapors leads to significant changes in optical absorption and fluorescence emission.

These results are in contrast with those obtained from both H-PB and J-PB films of **1** after exposure to saturated vapors of monotopic Lewis bases of nitrogen-containing VOCs (N-VOCs) [71]. For example, exposure of both H-PB (10 min exposure) and J-PB (5 min exposure) films to saturated vapors of propylamine (PA, 356,538 ppm at 23 °C), which is closely related to EDA since both molecules have approximately the same length, leads to the formation of red films, with new absorption bands between 450 and 540 nm, and a new strong orange emission band at 614 nm, stronger than that of J-PB or H-PB films (Figure S1). This is the common behavior of **1** towards monotopic N-VOCs which, after chemisorption, gives rise to a vapochromic and vapoluminescent response associated with the formation of monomeric adducts, always resulting in a new emission band, accompanied by a fluorescence enhancement (Figure S2).

### 2.2. Sensing Mechanism for Detection of EDA Vapors

Based on the above results, it appears that both H-PB and J-PB films of **1** respond discriminatively to vapors of the ditopic EDA compared to vapors of monoamines. This observed distinct optical behavior of PB films of **1** after chemisorption of EDA is likely a consequence of a different binding mode of ditopic species to H- or J-aggregates, with respect to monotopic species.

**Figure 2:**
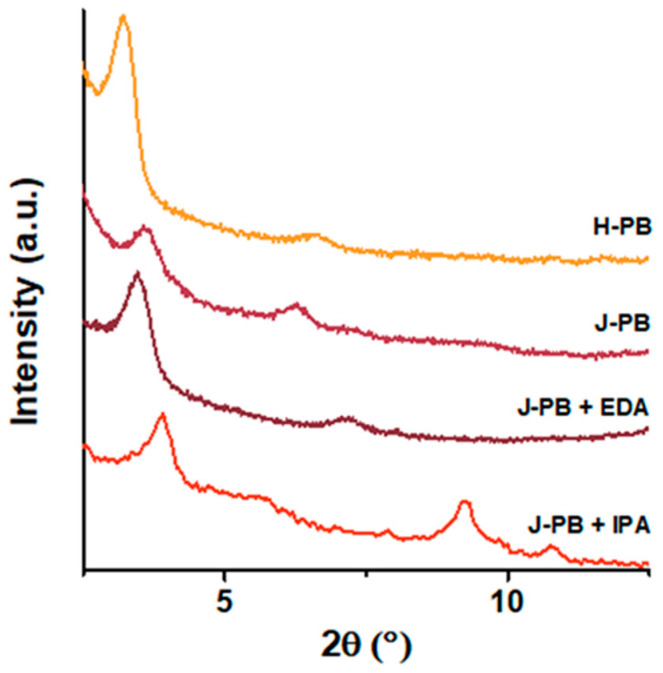
Comparison of low-angle XRD patterns for H-PB and J-PB films before and after exposure to saturated vapors of EDA and IPA. Identical XRD patterns, to J-PB + EDA, are obtained from H-PB films after exposure to saturated EDA vapors.

The XRD patterns (Figure 2) of H-PB or J-PB films after exposure to saturated EDA vapors, which are identical to each other, are very different from that recorded after exposure to saturated vapors of a monotopic amine, such as isopropylamine (IPA), thus indicating the formation of a different structure after chemisorption of the diamine. In particular, the XRD pattern shows the presence of a sharp peak at small angles (2θ = 3.45°) and a peak at 2θ = 7.15°, analogous to those observed for H-PB and J-PB aggregates, indicating the existence of a self-assembled structure with higher-symmetry [72,73], upon coordination of the diamine to complex **1**, compared to that obtained with the monotopic amine [63].

**Figure 3:**
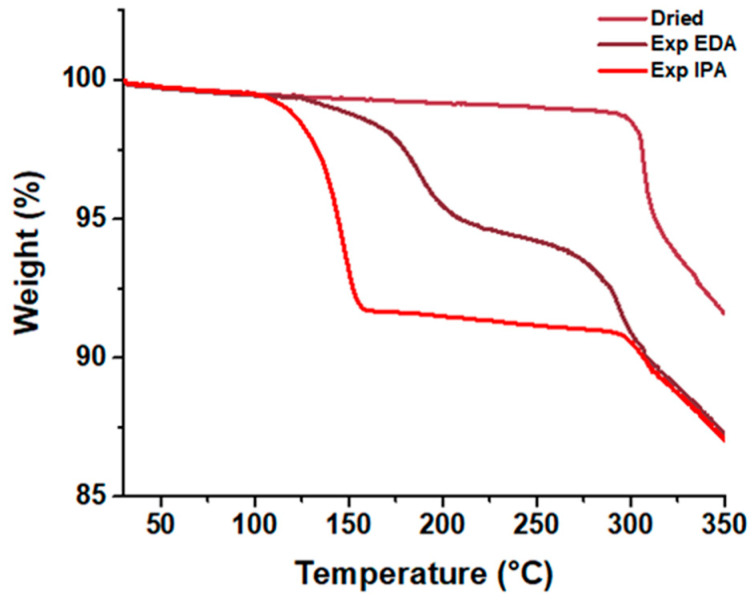
Thermogravimetric analysis of dried solid **1** and after exposure to saturated vapors of EDA and IPA.

These results suggest that both H- and J-aggregates, upon exposure to ditopic EDA base vapors, likely lead to the formation of intermolecular 2:1 adducts, (**1**)_2_·EDA, in a highly organized self-assembled structure. This hypothesis is demonstrated by thermogravimetric analysis (TGA). In fact, the TGA of a dried sample of **1** after exposure to saturated EDA vapors (Figure 3) shows a weight loss at ca. 220 °C of 4.5%, consistent with the EDA content for the (**1**)_2_·EDA adduct (4.0%). Instead, the thermogram of the dried solid **1** after exposure to saturated IPA vapors (Figure 3) shows quite different behavior, with a weight loss (7.7% loss) at a lower temperature (ca. 150 °C), consistent with the formation of the **1**·IPA adduct (7.62%) [63].

**Figure 4:**
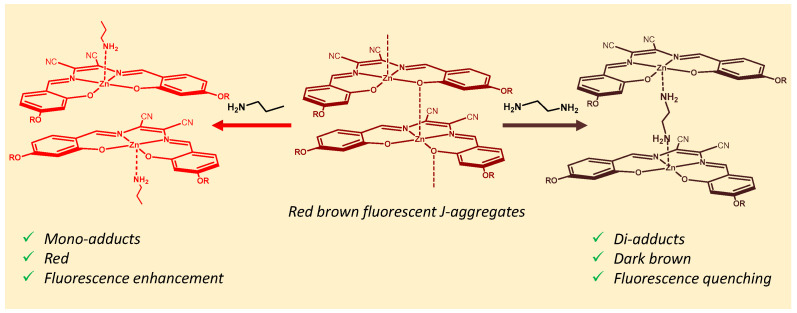
Schematic representation of the formation of mono- and di-adducts after chemisorption of primary aliphatic mono- or diamine vapors, respectively, to J-aggregates of **1**.

Overall, the formation of 2:1 intermolecular di-adducts upon chemisorption of diamine vapors, instead of 1:1 mono-adducts in the case of monoamines, accompanied by different structures, as evidenced by comparison of XRD patterns, are likely responsible for the different optical absorption and fluorescent emission behaviors of PB films of **1** toward diamine vapors compared to monoamines. Figure 4 shows a sketch of the discriminative behavior of J-aggregates of **1** after chemisorption of monotopic versus ditopic primary aliphatic amine vapors. The formation of mono-adducts leads to red films and a fluorescence enhancement, while the formation of di-adducts leads to dark-brown films and a fluorescence quenching.

### 2.3. Optical Response of PB Films Towards Other Volatile Diamines

**Figure 5:**
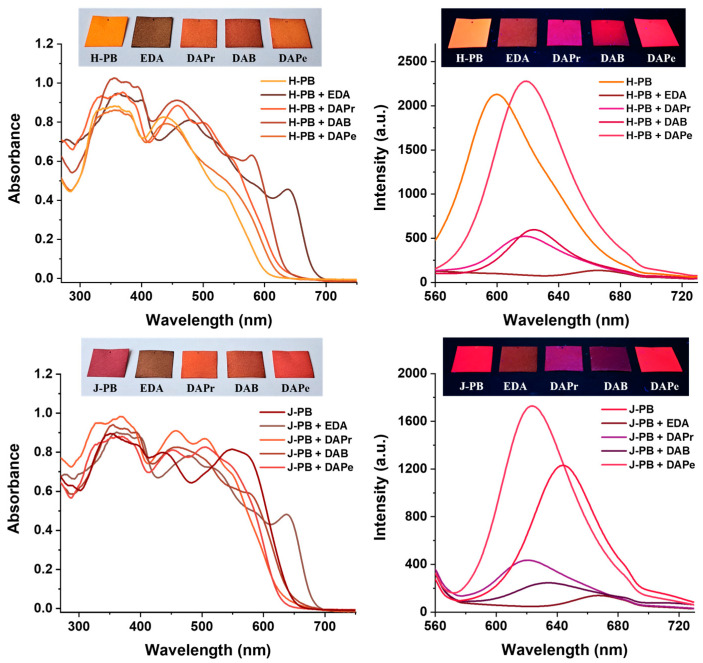
UV-vis reflectance (**left**) and fluorescence ((**right**); λ_exc_ = 520 nm) spectra of H-PB (**top**) and J-PB (**bottom**) films and after exposure to saturated vapors of the diamines involved. Inserts: photographic images of H-PB and J-PB films and after exposure to saturated vapors of the diamines involved under natural light (**left**) and 365 nm light (**right**).

The responsive behavior of PB films towards other volatile diamines was also investigated. In particular, the series of the following diamines, 1,3-diaminopropane (DAPr), 1,4-diaminobutane (DAB) or putrescine, and 1,5-diaminopentane (DAPe) or cadaverine was considered. H-PB and J-PB films were then exposed to saturated vapors of these volatile diamines and their optical absorption and fluorescence emission were compared to those obtained after exposure to EDA (Figure 5). Although for both H-PB and J-PB films, the changes in optical absorption and fluorescence emission appear less pronounced than those observed after exposure to saturated EDA vapors, each diamine exhibits a distinct behavior.

In particular, after exposure to saturated DAB vapors both H-PB and J-PB films turn brown, which is consistent with the formation of a new absorption band at longer wavelengths (579 nm), in addition to a broad absorption between 400 and 520 nm, associated with a significant quenching of their fluorescence emission at 600 nm and 644 nm and the formation of a new, weaker emission band at 617 nm and 633 nm, for H-PB and J-PB films, respectively. Instead, exposure to saturated DAPr vapors leads to the formation of red films, again with significant quenching of the fluorescence emission. Finally, cadaverine essentially behaves as a monotopic species since upon its exposure, similar to PA, a new strong fluorescence emission band at ca. 621 nm is observed in both H-PB and J-PB films. Therefore, these results suggest that the peculiar optical response of PB films to saturated EDA vapors, the formation of the new optical absorption band at 638 nm along with the appearance of a new very weak fluorescence emission at ca. 665 nm, allow to distinguish them from the other investigated diamines.

**Figure 6:**
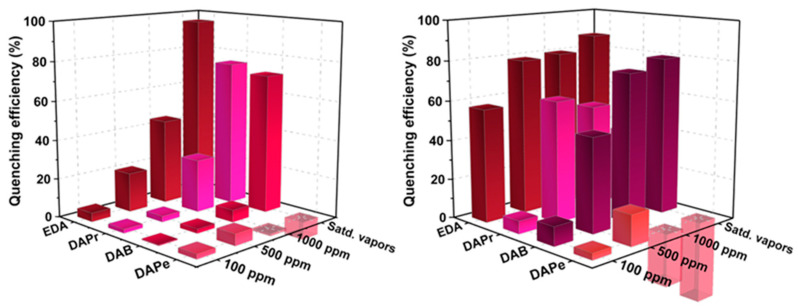
Fluorescence quenching efficiency at their emission maximum (λ_exc_ = 520 nm) of H-PB (**left**) and J-PB (**right**) films after exposure to various diamines to different vapor concentrations. Negative values imply an enhancement of fluorescence.

Although exposure of H-PB and J-PB films to saturated vapors of these volatile amines induces changes in both optical absorption and emission spectra, the latter appear more relevant for their detection. Therefore, the following discussion will mainly focus on the fluorescent response of H-PB and J-PB films. On the other hand, the relative variations in fluorescence along the diamine series are strongly dependent on the concentration of diamine vapors (Figures S3 and S4). In this regard, the fluorescence quenching efficiency (QE, calculated at the emission maxima by dividing the quenched fluorescence, I, by the initial fluorescence, I_0_, multiplying by 100, then subtracting from 100) [74] after exposure of H-PB and J-PB films to different vapor concentrations for the involved diamines is relevant to evaluate their fluorescence response (Figure 6). Thus, within the diamine series, H-PB and J-PB films are characterized by a different QE, more pronounced for the latter, whereas for the former a significant QE is evident at high vapor concentrations.

In particular, at saturated vapor concentrations, the H-PB films, with the exception of DAPe, exhibit a remarkable QE (up to 96% for EDA, 75% for DAPr, and 71% for DAB), while at lower vapor concentrations only EDA (20% at 500 ppm) and DAPr (27% at 1000 ppm) show an appreciable QE useful for their detection at such concentrations. Instead, J-PB films, again with the exception of DAPe, at vapor concentrations ≥ 500 ppm show more relevant QE values, close to saturation, EDA (79%), DAPr (61%), and DAB (48%). Furthermore, in the case of EDA a QE of 57% is observed even at lower vapor concentrations (100 ppm), while in these conditions the other diamines show small QEs (<10%). Therefore, J-PB films are useful for the selective detection of EDA at low vapor concentrations (<100 ppm).

### 2.4. Sensitive Detection of EDA Vapors

**Figure 7:**
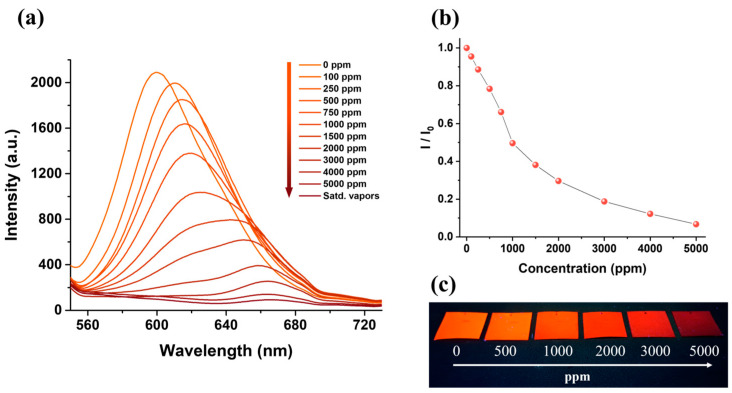
(**a**) Fluorescence spectra (λ_exc_ = 520 nm) of H-PB films after exposure to an increasing concentration of EDA vapors. Each spectrum was recorded in an independent experiment. (**b**) Fluorescence intensity ratio at the emission maxima as a function of the concentration of EDA vapors. I_0_ is the initial fluorescence. (**c**) Photographic images of H-PB films exposed to various concentrations of EDA vapors under 365 nm light.

The different fluorescence responses of H-PB and J-PB films as a function of the concentration of EDA vapors can be exploited for their detection. Thus, static exposure of H-films to increasing concentrations of EDA vapors (hundred ppm) leads to a gradual quenching of its fluorescence at 600 nm, approximately linearly up to 1000 ppm, whose emission maximum progressively shifts towards longer wavelengths up to 665 nm (Figure 7). Saturation occurs at EDA vapor concentrations above 3000 ppm, until an almost complete quenching of the fluorescence at about 5000 ppm (QE 93%). These results are relevant for the detection of the EDA to high vapor concentrations, from >100 ppm to 3000 ppm, the upper limit of response for H-PB films. Furthermore, they allow for the detection of EDA vapors at the immediately dangerous for life or health (IDLH) concentration (1000 ppm), as established by the National Institute for Occupational Safety and Health (NIOSH) [75].

**Figure 8:**
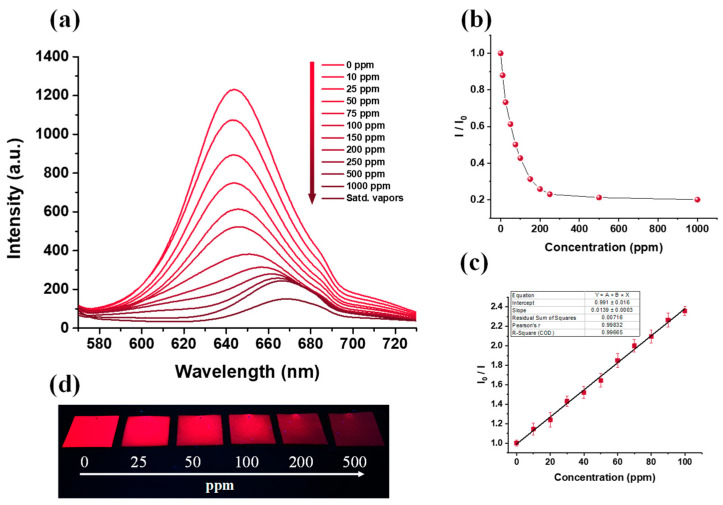
(**a**) Fluorescence spectra (λ_exc_ = 520 nm) of J-PB films after exposure to an increasing concentration of EDA vapors. Each spectrum was recorded in an independent experiment. (**b**) Fluorescence intensity ratio at the emission maxima as a function of the concentration of EDA vapors. I_0_ is the initial fluorescence. (**c**) Linear best fit in the linear dynamic range. (**d**) Photographic images of J-PB films exposed to various concentrations of EDA vapors under 365 nm light.

As anticipated, J-PB films are selectively responsive to lower vapor concentrations of EDA. Static exposure of J-films to increasing concentrations (tens of ppm) of EDA vapors, first leads to a progressive quenching of the emission band at 644 nm, up to 100 ppm, then at higher ppm concentrations the maximum shifts to 665 nm (Figure 8). Saturation occurs at EDA vapor concentrations above 200 ppm. Moreover, a linear dynamic range up to 100 ppm is achieved, with a good linearity coefficient (R^2^ = 0.9967), allowing to estimate a limit of detection (LOD) for EDA vapors down to 6.6 ppm. The latter is relevant because it is below the PEL of 10 ppm established by OSHA for EDA [7]. These results compare favorably with the few data on optical chemosensors reported in the literature for the selective and sensitive detection of EDA vapors [49,50], with the advantage of being a simple, cost-effective method for the preparation of PB films.

In summary, the combination of H-PB and J-PB vapoluminescent films enables discriminative detection of EDA vapors over a wide concentration range from tens to thousands ppm. This is very important to estimate the risk to human health caused by long and/or short exposure times in the presence of specific vapor concentrations of this substance. All these features make PB films a unique example of optical chemosensor for the detection of EDA vapors among those reported in the literature [35,36,49,50].

## 3. Materials and Methods

### 3.1. Materials and Physical Measurements

All the chemicals used were purchased from Sigma-Aldrich (Merk Life Science S.r.l., Milano, Italy) and used as received. Complex **1** was synthesized, purified, and characterized as previously reported [76]; it was dried at 140 °C in an oven for 30 min before use. UV-vis diffuse reflectance spectra on PB films were recorded with a JASCO V-750 spectrophotometer (Cremella, Italy). Fluorescence measurements were performed in a front-face configuration (incident angle of the excitation light 30°) with a JASCO FP-8200 spectrofluorometer equipped with a film holder, model FLH-809 (ex/em slits = 5/5 nm). TGA measurements were performed using a Mettler Toledo TGA2 (Mettler-Toledo S.p.A., Milano, Italy) and STARe software (https://www.mt.com/gb/en/home/products/Laboratory_Analytics_Browse/TA_Family_Browse/TA_software_browse.html, accessed on 15 December 2024), under prepurified nitrogen flow, fed into the working chamber at 50 sccm with a 5 °C/min heating/cooling rate at atmospheric pressure. Each solid sample was subjected to isothermal heating at 50 °C for 20 min under a nitrogen atmosphere before recording the thermogram. X-ray diffraction patterns on PB films were recorded in grazing incidence mode (0.5°) on a Smartlab Rigaku diffractometer (Rigaku, Tokyo, Japan) equipped with a rotating anode of Cu Kα radiation (λ = 1.5418 Å) operating at 45 kV and 200 mA.

### 3.2. Preparation of Paper-Based Films and Vapor Exposure Experiments

The dip-coating technique was used to obtain H-PB and J-PB films of the complex. Films were deposited onto Whatman-4 filter paper substrates, using a 5.0 × 10^−3^ M THF solution of the complex. Further details on the preparation of H-PB and J-PB films are given elsewhere [71]. This procedure ensures the optical uniformity and repeatability of films.

Exposure to saturated vapors of the involved volatile compounds was carried out in a sealed glass chamber, in which H-PB and J-PB films and the volatile liquid were placed inside. H-PB films were exposed at room temperature for 10 min in the case of EDA and 30 min for all other investigated diamines, while J-PB films were exposed for 5 min in the case of EDA and butylamine, and 10 min for the other volatile species.

Exposure of H-PB and J-PB films to defined concentrations of the studied diamines was carried out under static conditions. The experimental setup for the static exposure experiments and the details of the static liquid–gas distribution method [77,78] to calculate the desired ppm concentration of the involved VPAD are described elsewhere [71]. Exposure to specific concentrations of diamines was performed by injections into the sealed chamber (1.15 L), solutions of the involved VPAD in Et_2_O (b.p. 35.0 °C) with a concentration of 0.0473 M, allowing their complete evaporation. This concentration was chosen such that 1 µL of the 0.0473 M solution injected into the sealed chamber is equal to a concentration of 1 ppm (useful in the range from 1 to 100 ppm). For exposure to higher concentrations, appropriate volumes of pure diamine were injected into the sealed chamber. The exposure time was set at 60 min for each diamine in the case of H-PB films and 30 min for J-PB films, at room temperature (23 ± 3 °C) with a relative humidity of 45 ± 5%. This exposure time ensures that fluorescence intensity saturation is reached for all concentrations explored.

### 3.3. Calculation of the Limit of Detection

The limit of detection for EDA vapors was calculated in the dynamic linear range (0–100 ppm) with the following formula:

(1) $$LOD=K\times \frac{S_{b}}{S}$$

where *K* is a constant, *S_b_* is the standard deviation of the blank, and *S* is the slope derived from the linear fit curve. *S_b_* was estimated from over 120 replicate measurements, *n*, of the fluorescence intensity at λ_em_ = 644 nm, I_0*n*_, of the ratio I_0_/I_0*n*_, where I_0_ is the mean value of 120 replicates. The calibration curve was obtained from the plot of the ratio I_0_/I versus the concentration of the diamine added. Each point is related to the mean value obtained from at least three replicate measurements. Using Equation (1), with *K* = 3, *S_b_* = 0.031, and *S* = 0.014, a LOD value of 6.6 ppm was calculated

## 4. Conclusions

This contribution explores the distinctive features of H-PB and J-PB films of a Lewis acidic Zn(salen)-type complex upon chemisorption of vapors of ditopic VPADs versus those of monotopic volatile amines. While volatile monotopic Lewis bases give rise to mono-adducts accompanied by an enhancement of the fluorescence, VPADs, with the exception of DAPe, act as ditopic bases forming di-adducts with distinct optical properties, leading to fluorescence quenching. This different behavior allows to distinguish VPAD vapors from those of volatile monoamines, enabling their sensitive detection.

Among VPADs, H-PB, and J-PB films are more sensitive to EDA vapors. Thus, using J-PB films a LOD down to 6.6 ppm below the PEL for EDA (10 ppm) is obtained, with a dynamic linear range up to 100 ppm. On the other hand, H-PB films allow for the detection of EDA vapors at higher ppm concentrations (up to 3000 ppm) with a linearity of up to 1000 ppm, representing the IDLH concentration. Therefore, the combination of both H-PB and J-PB films of the Zn(salen)-type complex, represents a unique example for the sensitive and discriminative detection of EDA vapors in such a wide concentration range.

Overall, PB films represent a simple, disposable, and cost-effective optical chemosensor for the discriminative and sensitive in situ direct detection of mono- or diamine vapors.

## Data Availability

The data presented in this study are available on request from the corresponding author.

## Supplementary Materials

The following supporting information can be downloaded at: https://www.mdpi.com/article/10.3390/molecules29245947/s1, Figure S1: UV-vis reflectance and fluorescence spectra of H-PB and J-PB films after exposure to saturated EDA and PA vapors; Figure S2: Fluorescence spectra and fluorescence intensity ratio of J-PB films after exposure to saturated vapors of various monotopic N-VOCs and EDA; Figure S3: Fluorescence spectra of H-PB films and after exposure to various diamines to different vapor concentrations; Figure S4: Fluorescence spectra of J-PB films and after exposure to various diamines to different vapor concentrations.
