# Supplementary material for: Sensitive and Discriminative Fluorescent Detection of Volatile Primary Aliphatic Diamine Vapors from Monoamines
Source: Molecules. 2024 Dec 17;29(24):5947. doi: 10.3390/molecules29245947 (PMC11677194; doi:10.3390/molecules29245947)
Supplement: Supplementary file 1 [file molecules-29-05947-s001.zip › molecules-3356999-supplementary.pdf]

Supplementary Materials

Sensitive and Discriminative Fluorescent Detection of Volatile Primary Aliphatic Diamine Vapors from Monoamines

Agostino Attinà, Ivan Pietro Oliveri, Massimiliano Gaeta and Santo Di Bella *

Dipartimento di Scienze Chimiche, Università di Catania, Viale Andrea Doria, 6-I, 95125 Catania, Italy; agostino.attina@phd.unict.it (A.A); ivan.oliveri@unict.it (I.P.O.); massimiliano.gaeta@unict.it (M.G.)

*Correspondence: sdibella@unict.it

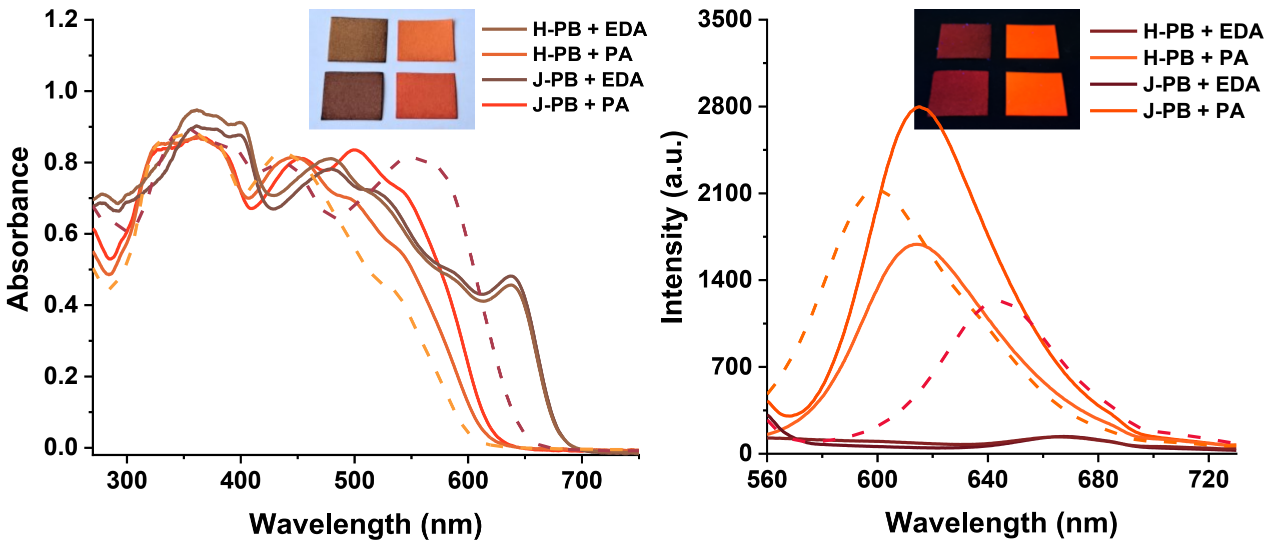

Figure S1. UV-vis reflectance (left) and fluorescence (right; λ_{exc} = 465 nm) spectra of H-PB and J-PB films after exposure to saturated EDA and PA vapors. Spectra of H-PB and J-PB films are reported for comparison (dashed lines). Inserts: photographic images of H-PB and J-PB films after exposure to saturated EDA and PA vapors under natural light (left) and 365 nm light (right).

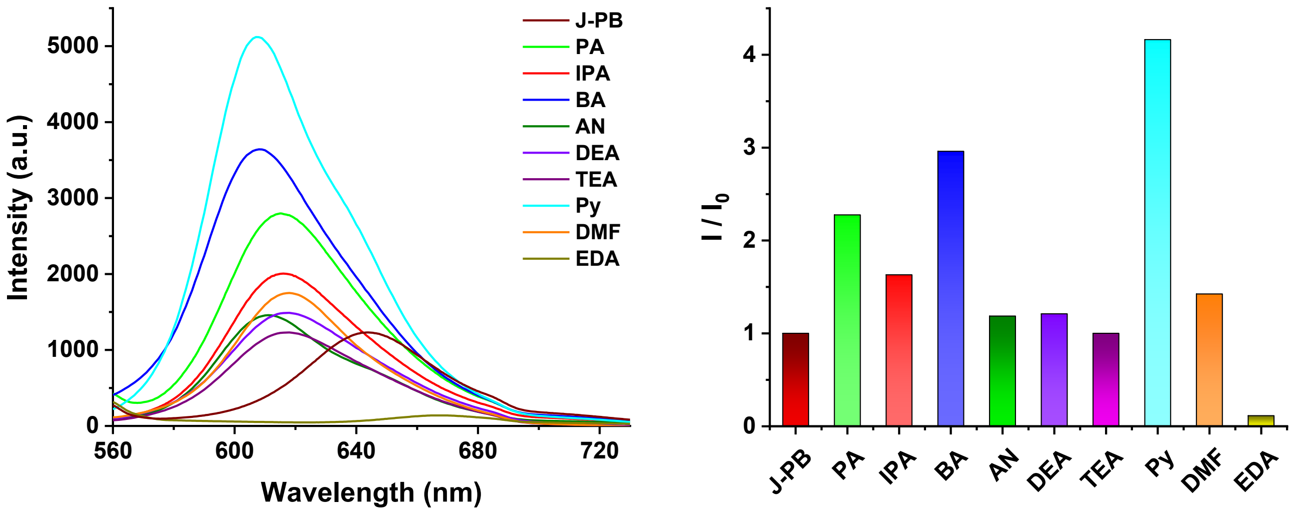

Figure S2. Fluorescence spectra (left; λ_{exc} = 520 nm) and fluorescence intensity ratio (right; λ_{exc} = 520 nm) of J-PB films after exposure to saturated vapors of various monotopic N-VOCs and EDA. I_0 and I is the fluorescence intensity at λ_{max} of J-PB films before and after exposure, respectively.

PA = propylamine; IPA = isopropylamine; BA = butylamine; AN = aniline; DEA = diethylamine; TEA = triethylamine; Py = pyridine; DMF = dimethylformamide.

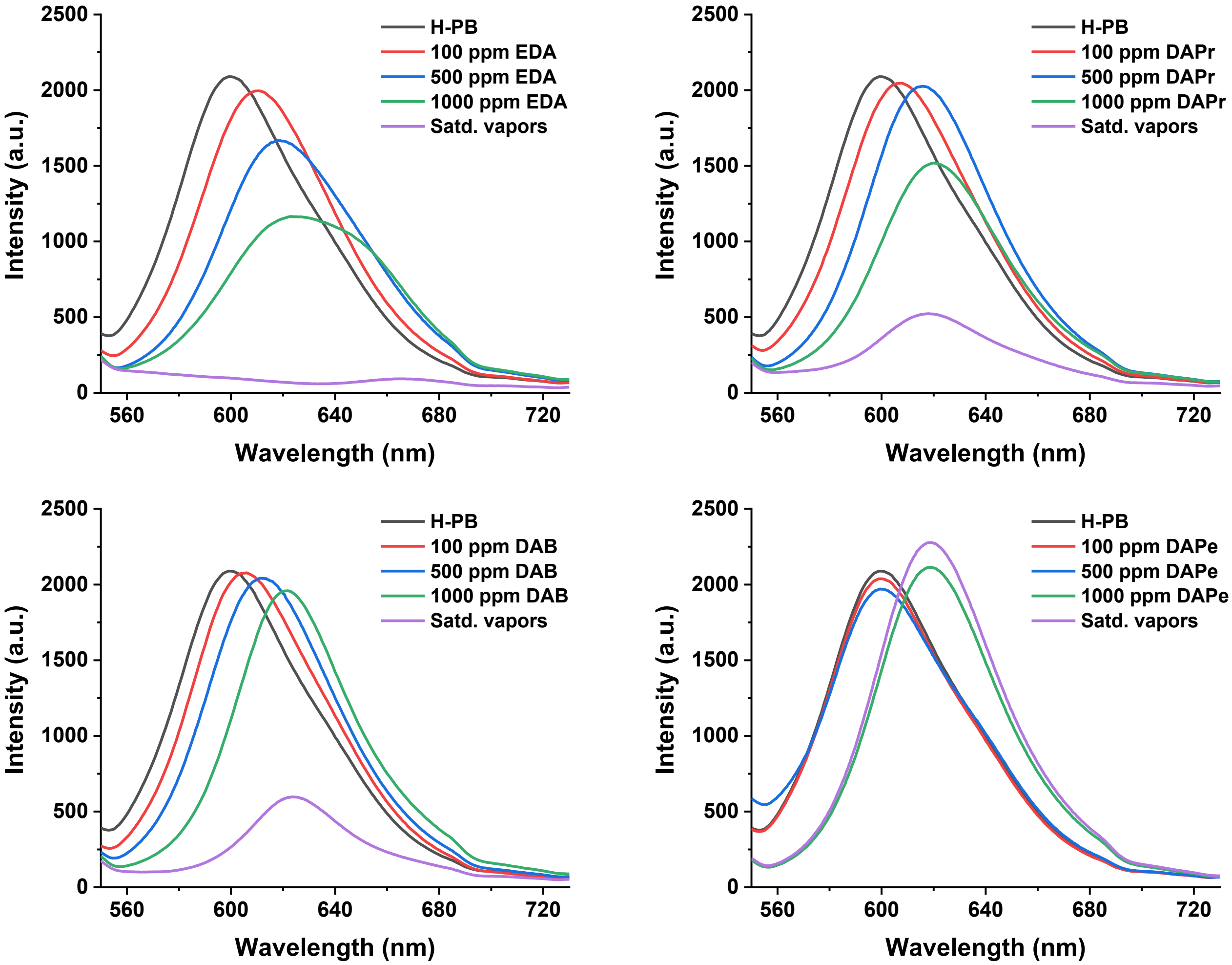

Figure S3. Fluorescence (λ_{exc} = 520 nm) spectra of H-PB films and after exposure to various diamines to different vapor concentrations.

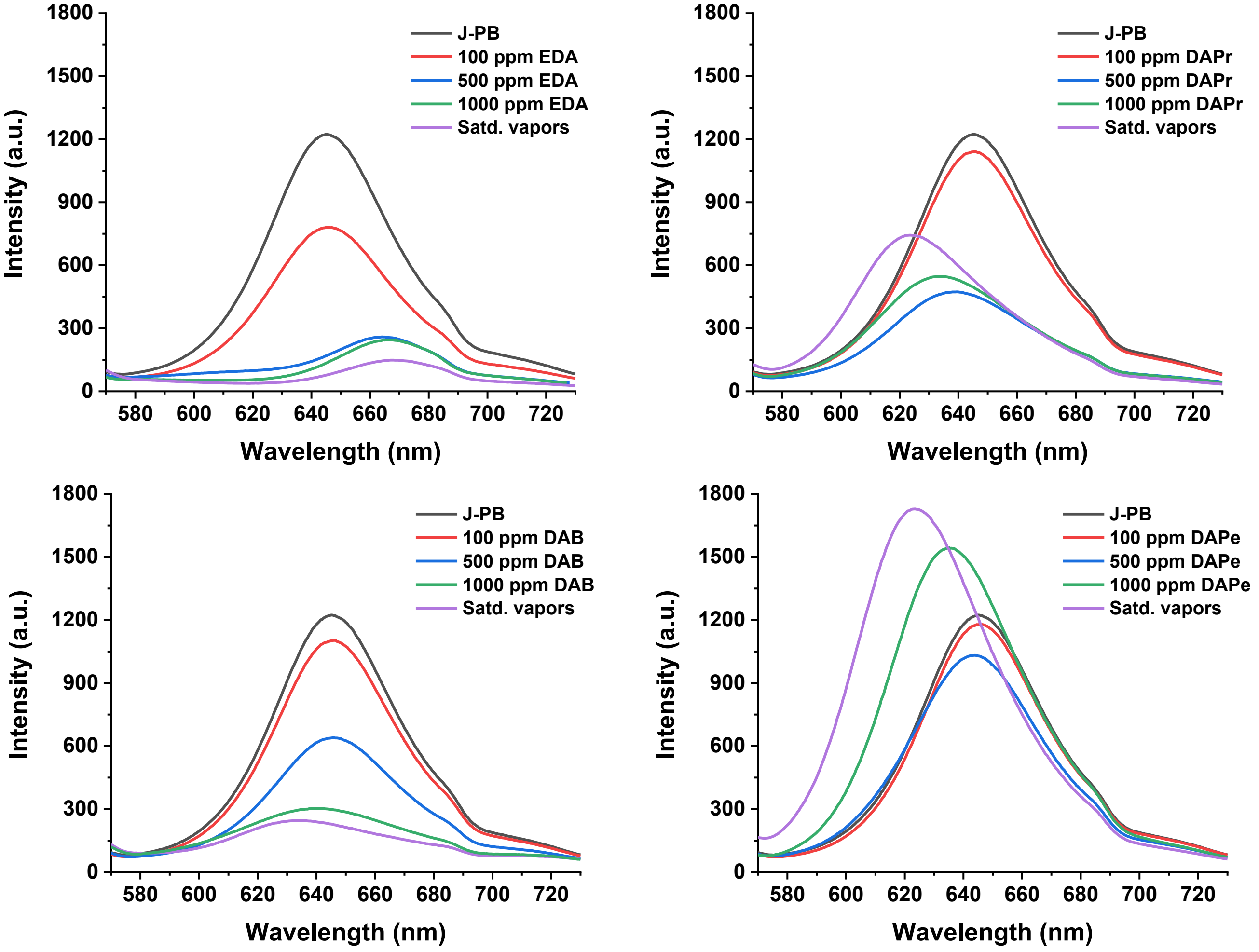

Figure S4. Fluorescence (λ_{exc} = 520 nm) spectra of J-PB films and after exposure to various diamines to different vapor concentrations.
